# Cold Agglutinin Autoimmune Hemolytic Anemia Revealing Undiagnosed Diffuse Large B-cell Lymphoma Following COVID-19 Vaccination

**DOI:** 10.7759/cureus.84833

**Published:** 2025-05-26

**Authors:** Faiza Humayun Khan, Ahsan Ayaz, Tamer Zahdeh

**Affiliations:** 1 Internal Medicine, Montefiore St. Luke's Cornwall, Newburgh, USA

**Keywords:** autoimmune disease, auto-immune hemolytic anemia, cold agglutunin hemolytic anemia, covid 19 vaccination, diffuse large b cell lymphoma (dlbcl)

## Abstract

Diffuse large B-cell lymphoma (DLBCL) is an aggressive non-Hodgkin lymphoma that can be associated with autoimmune hemolytic anemia (AIHA), including the rare cold agglutinin hemolytic anemia (CAHA) subtype. Emerging reports have also suggested a potential link between COVID-19 vaccination and the development of AIHA. We report a case of a 56-year-old male with a history of autoimmune diseases who developed symptomatic hemolytic anemia shortly after receiving an mRNA COVID-19 booster. Laboratory evaluation revealed hemolysis with a positive direct antiglobulin test for C3 and negative for IgG, consistent with CAHA. Imaging demonstrated hepatosplenomegaly and isolated lymphadenopathy, and subsequent biopsies confirmed the diagnosis of DLBCL. Although initiated on steroids for presumed autoimmune hemolysis, the patient’s limited response further supported CAHA secondary to underlying lymphoma. While COVID-19 vaccines are widely recognized as safe and effective, rare cases of post-vaccination AIHA have been reported. In this case, it remains unclear whether the CAHA was directly triggered by vaccination, unmasked by underlying DLBCL, or a coincidental occurrence. The temporal association observed highlights the complex interplay between immune activation, vaccination, and hematologic malignancy. This case underscores the importance of maintaining a broad differential diagnosis when evaluating hemolytic anemia following vaccination, particularly in patients with autoimmune backgrounds, and highlights the need for further research to better understand potential immunologic interactions among COVID-19 vaccination, AIHA, and occult lymphoma.

## Introduction

Autoimmune hemolytic anemia (AIHA) is an uncommon condition resulting from autoantibodies targeting the body’s own red blood cells. It is classified into several subtypes, including warm AIHA, cold agglutinin hemolytic anemia (CAHA), paroxysmal cold hemoglobinuria, mixed-type AIHA, and drug-induced AIHA [1]. Among other etiologies, AIHA has been associated with lymphoproliferative disorders, including Diffuse Large B-Cell Lymphoma (DLBCL) [1, 2]. In these cases, hemolysis may be an early indicator of an underlying malignancy, especially when no obvious infectious or autoimmune cause is identified. Beyond hematologic cancers, recent literature has reported a possible temporal association between Coronavirus Disease 2019 (COVID-19) vaccination and the onset of AIHA, raising questions about vaccine-induced immune activation as a potential trigger in susceptible individuals [3, 4]. We present a case of previously undiagnosed DLBCL manifesting as CAHA shortly after COVID-19 vaccination, highlighting the diagnostic complexity when immune-mediated cytopenia occurs in the post-vaccination setting.

## Case presentation

**Figure 1:**
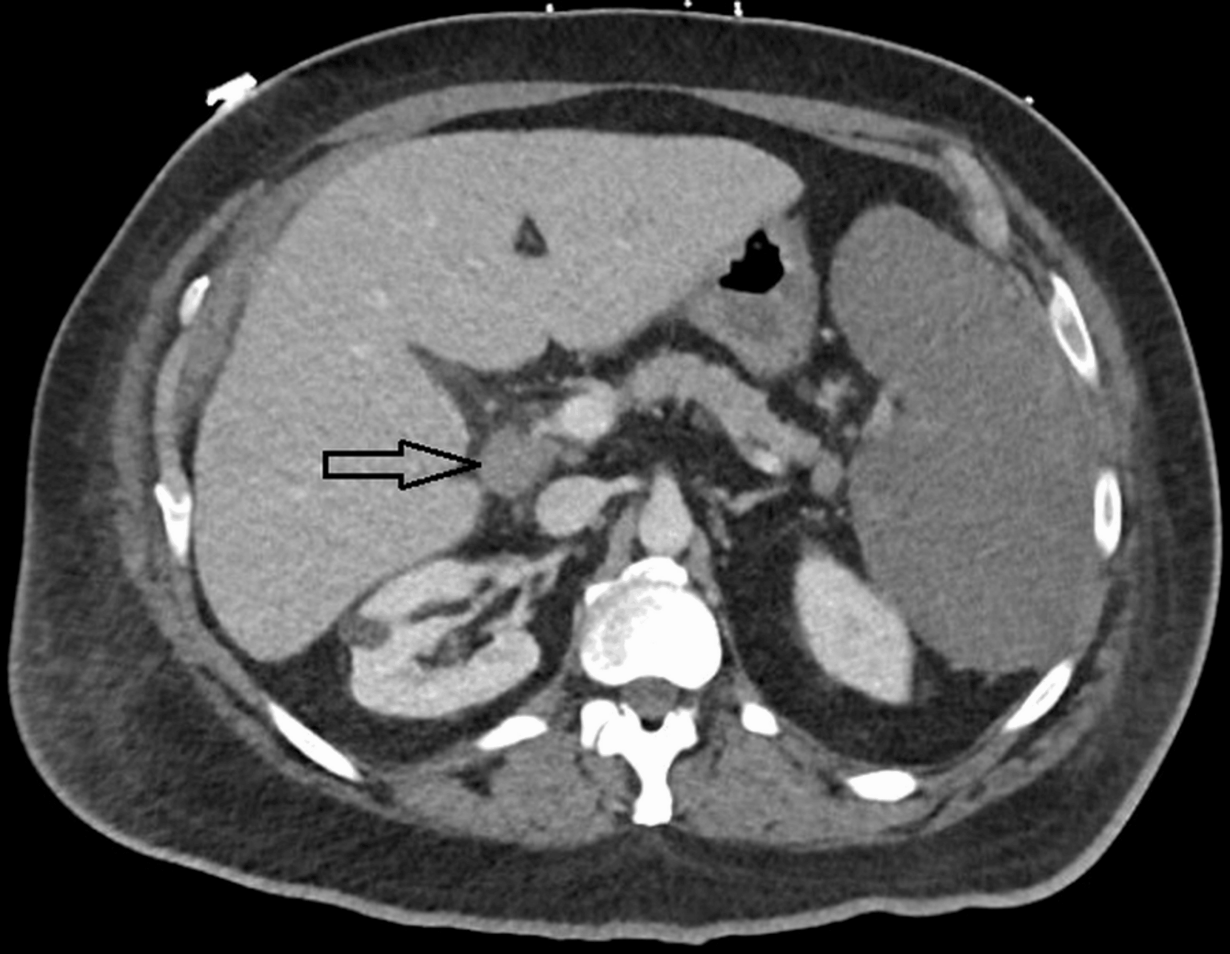
Computed tomography (CT) of the abdomen demonstrating hepatosplenomegaly and marked porta hepatis lymphadenopathy (arrow).

A 56-year-old male with a past medical history of vitiligo, Graves’ disease, and diabetes mellitus presented to the hospital with symptoms of lethargy, loss of appetite, night sweats, and 10-lb weight loss for 1 month. The patient reported that the onset of the above-mentioned symptoms started within a week of the mRNA booster vaccine for COVID-19. The physical exam was remarkable for pallor and jaundice. Laboratory workup revealed leukocytosis (White blood cell count 24 K/uL), anemia (Hemoglobin 8.0 g/dL), thrombocytosis (platelets 554 K/uL), mild transaminitis (alanine aminotransferase 88, aspartate aminotransferase 164, alkaline phosphatase 273), and hyperbilirubinemia (total bilirubin 11.2 mg/dL, direct bilirubin 8.3 mg/dL). Haptoglobin was <7.75 mg/dL, reticulocyte count was elevated (30.59%), and lactate dehydrogenase was 1423 U/L, all suggestive of hemolysis (Table 1). Acute phase reactants were elevated (C-reactive protein 14 mg/dL, ferritin 944 ng/mL). The direct antiglobulin test was positive for complement component 3 (C3) but negative for immunoglobulin G (IgG), a serologic pattern consistent with CAHA. Flow cytometry showed a high CD4/ CD8 positive T-Cell ratio of 17.23. Computed tomography (CT) of the abdomen and pelvis revealed hepatosplenomegaly with isolated lymphadenopathy in the porta hepatis (Figure 1). Further evaluation, including lymph node biopsy, demonstrated a mixed lymphoid population with occasional large atypical lymphoid cells. Bone marrow biopsy revealed hypercellular marrow (70-80%) with trilineage hematopoiesis and approximately 10% involvement by atypical large CD20+, BCL2+, BCL6+ atypical B-cells, negative for CD10, MUM1, CD30, P53, and Cyclin D1. CMYC expression was equivocal (~30%). FISH analysis demonstrated complex chromosomal abnormalities with polysomy in a small subset (2%) of cells, below the diagnostic threshold but supportive of cytogenetic instability. Flow cytometry showed no blast proliferation or aberrant myeloid markers. B-cells comprised 2.01% of marrow cells. Liver biopsy showed infiltration by large atypical lymphoid cells, consistent with involvement by the patient’s DLBCL. These histopathologic and immunophenotypic findings, along with the liver biopsy showing infiltration by large atypical lymphoid cells, collectively confirm the diagnosis of DLBCL.

**Table 1:** Laboratory values on admission.

Parameter	Result	Units	Reference Range
White Blood Cells (WBC)	24.0	K/μL	4.0–10.0 K/μL
Hemoglobin (Hgb)	8.0	g/dL	Female: 12.0–16.0 g/dL
Platelets	554	K/μL	150–450 K/μL
ALT (Alanine Aminotransferase)	88.0	U/L	10–40 U/L
AST (Aspartate Aminotransferase)	164.0	U/L	10–40 U/L
ALP (Alkaline Phosphatase)	273.0	U/L	30–120 U/L
Total Bilirubin	11.2	mg/dL	0.3–1.0 mg/dL
Direct Bilirubin	8.3	mg/dL	0.1–0.3 mg/dL
Haptoglobin	<7.75	mg/dL	30–200 mg/dL
Reticulocyte Count	30.59	%	0.5–1.5%
LDH (Lactate Dehydrogenase)	1423.0	U/L	100–200 U/L
CRP (C-Reactive Protein)	14.0	mg/dL	<1.0 mg/dL
Ferritin	944.0	ng/mL	Female: 12–150 ng/mL
CD4/CD8 Ratio	17.23	Ratio	0.8–4.5

Broad-spectrum antibiotics were initiated for possible infection but later discontinued following negative infectious workup, including blood culture, urinalysis, and respiratory and urine cultures. The patient was started on prednisone (1 mg/kg) for hemolytic anemia. Lack of laboratory improvement with steroid therapy further supported the diagnosis of CAHA. Patient’s hemoglobin dropped to 6.9 g/dl during hospitalization and required 1 unit of packed red blood cells transfusion. He was later discharged in stable condition with plans for oncology follow-up to initiate treatment for DLBCL; however, specific details regarding the appointment or the intended oncology provider were unavailable, suggesting he may have sought care elsewhere.

## Discussion

DLBCL, the most common and aggressive subtype of non-Hodgkin lymphoma, is characterized by the rapid proliferation of malignant B lymphocytes [5]. In rare cases, DLBCL can be associated with AIHA, either as a paraneoplastic manifestation or due to immune dysregulation driven by the malignancy [2]. The patient’s history of vitiligo and Graves’ disease reflects an underlying predisposition to autoimmune disorders [6]. Although COVID-19 vaccines have been proven safe and effective through large-scale clinical trials, careful monitoring for rare adverse effects remains important, especially in individuals with pre-existing autoimmune conditions. Notably, there have been reports linking COVID-19 vaccination to the onset of AIHA [3, 4]. Though rare, post-vaccination autoimmune events have raised concern, particularly in individuals with underlying autoimmune tendencies, as seen in our patient. Molecular mimicry between SARS-CoV-2 and human antigens has been proposed as a potential mechanism, possibly contributing to autoimmune responses after infection or vaccination [7]. CAHA is primarily a complement-mediated process, where IgM antibodies bind to red blood cells at low temperatures, activating the classical complement pathway and leading to hemolysis, mainly in the liver [8]. Steroids are generally ineffective in CAHA because they do not significantly inhibit complement activation, unlike their role in warm AIHA, where IgG-mediated phagocytosis predominates [8]. This limited response to steroids further supports the diagnosis of CAHA in the setting of underlying lymphoma.

In our case, it remains uncertain whether the AIHA was primarily related to the underlying DLBCL or potentially triggered by the immune activation associated with the COVID-19 booster. Of note, CAHA, a less common subtype of AIHA, has also been infrequently associated with DLBCL [9, 10]. The manifestation of our patient’s DLBCL and AIHA was precipitated and subsequently unveiled following the administration of the COVID-19 booster vaccine. This phenomenon underscores the complex interplay between malignancy, immune response, and vaccination. Numerous case reports have linked COVID-19 vaccination with lymphoproliferative disorders [11-13]. While this case highlights a potential association between COVID-19 vaccination, AIHA, and underlying lymphoma, it does not establish causality. Nevertheless, it emphasizes the importance of clinical vigilance, especially in patients with autoimmune backgrounds or unexplained cytopenias post-vaccination. Continued research, pharmacovigilance, and case reporting are essential to better understand the immunologic interplay and identify potential risk factors in susceptible individuals.

## Conclusions

This case highlights the need for a broad differential diagnosis when assessing hemolytic anemia, especially in patients with systemic symptoms and recent immune stimulation, such as vaccination. Although AIHA may occur as a primary autoimmune disorder, it can also serve as a paraneoplastic manifestation of malignancy, as demonstrated by CAHA secondary to occult DLBCL in this patient. Given the essential role of vaccines, clinicians should remain vigilant for rare hematologic complications, particularly in individuals with autoimmune predispositions. Further investigation is warranted to clarify the immunologic pathways linking vaccination, autoimmunity, and malignancy.
